# Multimodal Imaging of Diabetic Mastopathy: A 62‐Case Study With Ultrasound‐Based Classification and Diagnostic Accuracy

**DOI:** 10.1155/ijbc/7449526

**Published:** 2026-05-05

**Authors:** Yilin Xu, Yingxue Wang, Xiaokang Li, Yaqing Li, Xuejing Liu, Yuzhe Zhao, Ying Zhu, Hong Lu

**Affiliations:** ^1^ Department of Breast Imaging, Tianjin Medical University Cancer Institute and Hospital, National Clinical Research Center for Cancer, Tianjin′s Clinical Research Center for Cancer, Key Laboratory of Breast Cancer Prevention and Therapy, Tianjin Medical University, Ministry of Education, Key Laboratory of Cancer Prevention and Therapy, Tianjin, China, tmucih.com; ^2^ Department of Breast Pathology, Tianjin Medical University Cancer Institute and Hospital, National Clinical Research Center for Cancer, Tianjin′s Clinical Research Center for Cancer, Key Laboratory of Breast Cancer Prevention and Therapy, Tianjin Medical University, Ministry of Education, Tianjin, China, tmucih.com; ^3^ Physical Examination Center, Qiqihar Hospital of Traditional Chinese Medicine, Qiqihar, China

**Keywords:** breast MRI, diabetic mastopathy, mammography, multimodal imaging, ultrasound

## Abstract

**Purpose:**

The purpose of this study is to investigate the application of multimodal imaging technology in diagnosing diabetic mastopathy (DM).

**Methoods:**

This study retrospectively analyzed ultrasound, mammography, and MRI findings of pathologically confirmed DM from October 2014 to October 2024.

**Results:**

Ultrasonography was performed on all 62 patients with DM, identifying a total of 67 lesions. The ultrasound findings were classified into four types: Type I presented as a focal thickening and bulging of the gland with a mix of high and low echoes (42/67, 62.7%); Type II showed focal or overall hypoechoic areas with indistinct margins (19/67, 28.3%); Type III exhibited diffuse hypoechoicity in the lesion area, poorly visualized internal structures, and markedly attenuated posterior echoes (4/67, 6.0%); and Type IV consisted of slightly hypoechoic areas (2/67, 3.0%). The overall diagnostic accuracy of ultrasound was 58.2%. Fifty‐one patients with a total of 58 lesions underwent mammography. Eighteen lesions (31.0%) were negative on mammography, whereas 32 lesions (55.2%) displayed asymmetric density. The diagnostic accuracy of mammography was 69%. Five patients with five lesions underwent MRI, which revealed nonmass‐like inhomogeneous progressive enhancement, isointensity and hypointensity in T2‐weighted images, insignificant or slight hyperintensity in diffusion‐weighted imaging, and no significant decrease in apparent diffusion coefficient value, yielding a diagnostic accuracy of 80%.

**Conclusion:**

The ultrasound characteristics of DM were marked by nonmass‐type lesions with distinctive echogenic and structural features. Mammography demonstrated insignificant or nonspecific asymmetric densities without suspicious calcifications or structural distortions. Breast MRI indicated nonmassive lesions exhibiting benign features based on hemodynamic parameters and diffusion‐weighted imaging. The combined application of multimodal imaging enhanced diagnostic accuracy, particularly when incorporated with patient history.

## 1. Introduction

Diabetic mastopathy (DM) is a rare benign breast disease, accounting for less than 1% of all benign breast lesions [1, 2]. It is more common in long‐term diabetic patients, especially those with Type 2 diabetes and insulin‐dependent diabetes. Most studies on DM are case studies, with the largest case series including only 34 cases [3]. Currently, guidelines for the prevention and treatment of DM are not available. Some suggest that conservative treatment is advisable once malignancy has been ruled out, rather than opting for surgical biopsy or excision [4, 5]. Numerous studies [6, 7] have highlighted the difficulty in distinguishing DM from breast cancer through clinical examination and imaging. DM is highly prone to misdiagnosis during clinical management. Misdiagnosing breast cancer as DM delays cancer treatment, whereas misdiagnosing DM as breast cancer leads to unnecessary surgery or chemotherapy, resulting in overtreatment. Additionally, due to inconclusive imaging findings, patients may undergo multiple needle biopsies and surgical biopsies, increasing infection risks, delaying diagnosis and treatment, and imposing significant psychological and financial burdens on patients. Some studies have indicated that DM may resolve on its own, but it shows a high rate of postoperative recurrence [6, 8]. Improving the diagnosis rate of DM, standardizing treatment protocols, and establishing a well‐developed follow‐up plan are crucial for the management of DM.

Moreover, no correlation has been found between the imaging manifestations of DM and its pathological features. The objective of this study is to investigate the diagnostic utility of multimodal imaging techniques, including ultrasound, mammography, and MRI, in identifying DM, aiming to provide a reference for clinical treatment option selection.

## 2. Materials and Methods

### 2.1. Study Population

Our retrospective study was approved by our medical ethics committee and was conducted in accordance with the Health Insurance Portability and Accountability Act (HIPAA). Our medical ethics committee waived the requirement to obtain informed consent. All images were anonymized before processing. DM lesions seen at our hospital from October 2014 to October 2024 and confirmed by pathology were retrospectively analyzed. All patients were female, aged 30–78 years with a mean age of 62 years. A total of 71 lesions were identified in 62 patients, and 67 lesions were confirmed by puncture or surgical pathology. Eight cases had bilateral breast onset. Six patients had lesions found on the contralateral side after surgery for carcinoma on one side of the breast. Two patients presented with recurrent lesions, and both two episodes were included in the study.

### 2.2. Examination Methods

Breast ultrasonography was performed using a GE LOGIQ E9 diagnostic ultrasound machine, and the probe was selected to be a 6–15‐MHz variable‐frequency line‐array probe. We performed diagnostic ultrasonography on all patients. Patients were routinely placed in the supine position, with both upper limbs naturally raised to the top of the head, to fully expose the bilateral breasts, and were subjected to multisection two‐dimensional imaging sweeps and color Doppler examinations, among others. Mammography was applied to the LORAD Selenia fully digital mammography machine of HOLOGIC Company, United States, and diagnostic mammography was routinely performed, that is, bilateral cephalocaudal (craniocaudal, CC) and mediolateral oblique (MLO) photography. The exposure mode was typically set to AUTO‐TIME, and the appropriate compressor was selected based on the lesion location and breast size. Breast MRI was conducted using the GE 1.5T Signa Infinity EXCITE II and GE 3.0T Discovery magnetic resonance scanners, equipped with four‐channel breast‐specific phased array surface coils, with the patient positioned prone and both breasts allowed to sag. Following routine three‐plane localization scanning, plain scanning was performed with fast spin echo (FSE) T1WI (TR 700 ms, TE 10 ms), T2WI fat suppression (TR 4500 ms, TE 85 ms) in sagittal scanning of the affected breasts and in a transverse axial position, utilizing a slice thickness of 5 mm and a slice interval of 0.5 mm, with a matrix of 384 × 224 and NEX = 2. Diffusion‐weighted imaging (DWI) was conducted using a single excitation spin‐echo sequence (TR 6300 ms, TE 64 ms, matrix 128 × 128, slice thickness 5 mm, slice interval 0.5 mm, NEX = 4), with diffusion sensitivity values (b) set at 0 and 500 s/mm^2^. DCE‐MRI was performed with a breast‐optimized parallel acquisition of a 3D fast gradient echo sequence for bilateral sagittal volume imaging of the breast (VIBRANT). The mask was scanned first, followed by a bolus injection of the contrast agent Gd‐DTPA, administered via a high‐pressure syringe through the dorsal vein of the hand at a dose of 0.2 mL/kg and a flow rate of 2.0 mL/s. An equal amount of saline was injected immediately after completing the contrast injection, and eight or five‐time phases were scanned continuously, with a single‐phase scanning time of 60–100 s. The scanning parameters included TR 6.1 ms, TE 2.9 ms, inversion angle of 15°, matrix 256 × 128, FOV 26 × 26 cm, slice thickness 1.8 mm, and NEX 1. Finally, the transverse‐axial‐plane VIBRANT sequence scan of the delayed time phase was conducted.

### 2.3. Imaging Analysis

Two experienced breast imaging radiologists analyzed the lesions according to the 2013 BI‐RADS criteria. Discrepancies were resolved through discussion. Ultrasound analysis included lesion type (mass, nonmass, architectural distortion, ductal dilatation, etc.), margin, shape, internal echo and structural characteristics, surrounding tissue, posterior echo changes, color Doppler flow, and elastography. Mammography analysis included breast density type (fatty, scattered fibroglandular, heterogeneously dense, and extremely dense), presence of calcifications, mass characteristics (shape, margin, and density), architectural distortion, asymmetric, and other accompanying findings. MRI analysis included precontrast, dynamic contrast‐enhanced, DWI, and ADC values. Time‐intensity curves (TIC) were plotted for regions of interest (ROI) in the most enhancing areas of the lesions.

In the dynamic enhancement scanning of various phase images, we selected the parenchymal part of the tumor lesion and the slice with significant enhancement to define the ROI and plotted the time‐signal intensity curve (TIC). The observation indexes included the type of lesion (mass and nonmass), where we assessed the mass‐type lesion′s shape and margin and the nonmass‐type lesion′s distribution and internal enhancement characteristics.

### 2.4. Pathological Analysis

The specimens were fixed in a 10% neutral formalin solution, dehydrated, embedded in paraffin, sectioned into 5‐*μ*m sections, and stained with HE. The pathological features of DM include lymphocytic infiltration dominated by mature B‐cells around small vessels, lobules, and ducts, as well as dense interstitial fibrosis with lobular atrophy [9].

## 3. Results

### 3.1. Clinical Characteristics

All patients were female, aged 30–78 years, with an average age of 62 years. Sixty‐two patients had a total of 71 lesions, with 67 lesions confirmed by core needle biopsy or surgical pathology. The average lesion size was 3.0 cm (range: 1.3–7.7 cm). Eight patients had bilateral lesions. Six patients had a history of breast cancer in one breast and developed lesions in the contralateral breast. Two patients had recurrent lesions, with both episodes included in the study. Most lesions (63/67, 94.0%) were palpable, with 9 presenting as hard, thickened areas, 5 as firm, mobile masses, and 49 as hard, irregular, poorly mobile masses.

Tracing the menopausal status of the patients from whom the 67 lesions originated, 17 lesions originated from premenopausal patients (17/67 25.4%), and 50 lesions originated from postmenopausal patients (50/67 74.6%). Tracing the history of diabetes mellitus in the patients from whom the 67 lesions originated, 62 lesions (62/67, 92.5%) originated from patients with Type 2 diabetes mellitus, and 5 lesions (5/67 7.5%) originated from patients with Type 1 diabetes mellitus. The history of diabetes mellitus in the 62 patients included in the study ranged from 2 to 35 years with a mean of 15 years; 15 patients (15/62 24.2%) were treated with oral hypoglycemic agents and the remaining 46 patients (46/62 74.2%) with insulin, and a patient with one lesion (1/62 1.6%) presented with an admission fasting glucose value of 7.01 mmol/L (reference interval: 4.1–5.9 mmol/L). Five patients had diabetic complications (four with diabetic retinopathy and one with diabetic nephropathy), and eight had autoimmune diseases.

### 3.2. Ultrasound Manifestations

All 67 lesions were nonmass lesions with indistinct margins and heterogeneous internal echoes, absence of solid internal echogenicity, and no occupying effect. No cystic areas, ductal dilatation, calcifications, or structural distortions were observed. Posterior acoustic shadowing was seen in 29 lesions (43.3%). Color Doppler ultrasound showed no significant blood flow in 46 lesions, with 21 showing punctate or marginal blood flow. Elastography was performed in 47 lesions (we used the Tsukuba elastography scoring system), with 22 scoring 4, 18 scoring 2, and 7 scoring 3. No axillary lymphadenopathy was observed. Ultrasound BI‐RADS categories were 3 (8/67, 11.9%), 4A (31/67, 46.3%), and 4B or higher (28/67, 41.8%). Using BI‐RADS 3 and 4A as benign, the diagnostic accuracy was 58.2%.

**Figure 1:**
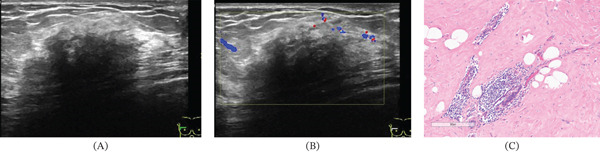
(A–B) Ultrasound revealed Type III diffuse hypoechoicity in the upper right breast, where the internal structures were poorly displayed. The posterior echoes were significantly attenuated, and marginal punctate blood flow signals were observed. (C) Micrographs (HE, ×200) demonstrated lobular atrophy of the breast, with a large number of lymphocytes infiltrating around the remaining lobular ducts, and interstitial fibroconnective tissue exhibiting significant proliferation.

The internal echogenicity and structural characteristics observed in the ultrasound imaging of DM were distinctive and can be categorized into four types: Type I, characterized by focal thickening and expansion of the gland along with mixed high and low internal echogenicity (42/67, or 62.7%); Type II, marked by focal or diffuse hypoechoic areas with indistinct margins (19/67, or 28.3%); Type III, distinguished by diffuse hypoechogenicity within the lesion, poorly visualized internal structures, and pronounced posterior acoustic shadowing (4/67, or 6.0%)(Figure 1); and Type IV, which presented with slightly hyperechoic areas (2/67, or 3.0%).

### 3.3. Mammogram Manifestations

**Figure 2:**
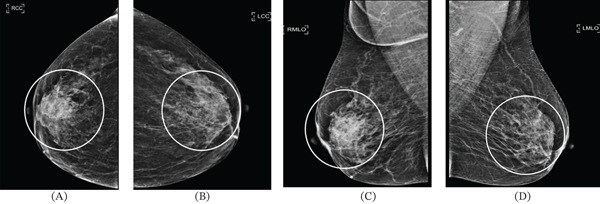
(A–D) Mammograms indicated focal asymmetry in the upper right breast, with no suspicious calcifications or structural distortions noted. We can see the asymmetry of the patient′s glands in both breasts through the portion of the figure labeled by the circle.

Fifty‐one patients with 58 lesions underwent mammography. Most patients had heterogeneously dense breasts (47/51, 45.5%), with one having extremely dense breasts (1/51, 36.4%) and three having scattered fibroglandular density (3/51, 18.2%). Eighteen lesions (31.0%) were negative on mammography, 32 (55.2%) showed focal asymmetry, and 8 (13.8%) had a slight architectural distortion. Two lesions showed round or indistinct calcifications. There are no suspicious calcifications or structural distortions in all lesions (Figure 2). Mammography BI‐RADS categories were 3 or lower (18/58, 31.0%), 4A (22/58, 37.9%), and 4B or higher (18/58, 31.0%). Using BI‐RADS 3 and 4A as benign, the diagnostic accuracy was 69%. Two cases were benign on ultrasound but malignant on mammography, and eight cases were malignant on ultrasound but benign on mammography. The remaining cases had consistent BI‐RADS classifications between the two modalities.

### 3.4. MRI Manifestations

**Figure 3:**
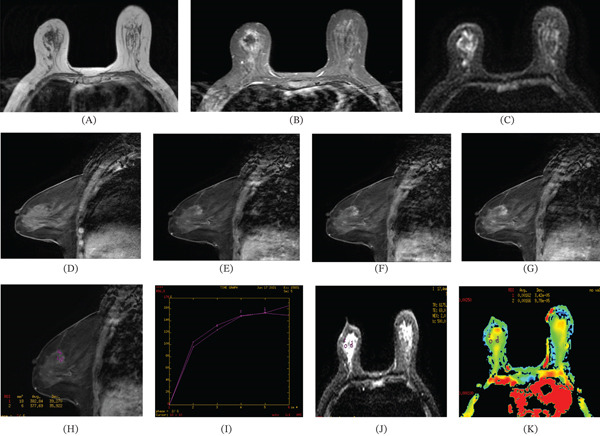
(A–C) T1‐weighted imaging showed isointensity, fat‐suppressed T2‐weighted imaging revealed hypointensity, and no abnormal hyperintensity was apparent in diffusion‐weighted imaging. (D–G) Pre‐enhancement and postenhancement Stages 1, 2, and 5, respectively, exhibited nonspecific patchy enhancement, characterized by low enhancement in the early stage and gradual enhancement thereafter. (H–I) Time‐intensity curves of Type I. (J–K) b‐Values of 500 s/mm^2^, an ADC value of 1.62–1.66 × 10^−3^ mm^2^/s, and no significant reduction was observed.

Five lesions in five patients underwent MRI (Figure 3). All lesions showed isointense signal on T1WI (5/5, 100.0%), with three showing isointense signal on fat‐suppressed T2WI (3/5, 60.0%) and two showing hypointense signal (2/5, 40.0%). DWI showed no significant signal in two lesions (2/5, 40.0%), with three showing a slightly high signal and no significant decrease in ADC values (3/5, 60.0%). All lesions showed nonmass‐like uneven progressive enhancement on dynamic contrast‐enhanced MRI, with Type I TIC (5/5, 100.0%), with no signs of malignancy such as focal or nodular, ductal or segmental enhancement. BI‐RADS categories were 3 (2/5, 40.0%), 4A (2/5, 40.0%), and 4B (1/5, 20.0%). Using BI‐RADS 3 and 4A as benign, the diagnostic accuracy was 80%.

## 4. Discussion

DM was first reported by Sloer and Khardori in 1984 as a rare benign fibrotic disease with unclear etiology and pathophysiology [10]. There are currently no effective diagnostic or treatment methods. DM was initially thought to be commonly found in patients with Type I diabetes [10], However, we found that the vast majority of our patients had Type II diabetes (62/67, 92.5%), consistent with some published reports [11, 12], This finding challenges the hypothesis linking DM to specific diabetes subtypes. Additionally, among the 62 patients, 8 had concomitant autoimmune diseases, supporting a potential association between diabetes mellitus and immune dysfunction.

Currently, there are no effective diagnostic methods for DM, which often leads to misdiagnosis and unnecessary surgical interventions during clinical diagnosis and treatment. A 2012 study on 34 DM patients found that 85.3% of diabetic patients had clinically palpable breast masses [3]. Given that distinguishing DM from BC is often challenging, pathological examination is frequently necessary for diagnosing DM. Due to the dense fibrotic components of DM, fine‐needle aspiration may result in insufficient sample volume. Ultrasound‐Guided Core Needle Biopsy (US‐CNB) is a useful tool for diagnosing DM [11]; however, some lesions still require additional biopsy procedures for definitive diagnosis [8, 13]. Surgical excision can completely remove the lesion but may stimulate disease progression, with a recurrence rate as high as 21.25%. Recurrences often occur in situ and frequently involve more breast tissue [14]. Repeated invasive procedures often impose significant psychological and financial burdens on patients, carry risks of stimulating lesions, and may delay diagnosis. However, pathological biopsy still remains the primary method for confirming most cases of DM. In this study, all 67 lesions underwent pathological examination.

Previous reports suggest that DM ultrasound features are similar to breast cancer and are often classified as BI‐RADS 4 and above [2, 15, 16]. Common ultrasound features of DM include hypoechoic masses with irregular shapes, unclear margins, parallel to the chest wall, posterior echo attenuation, no spiculation or lobulation, no significant blood flow, and no axillary lymphadenopathy [17, 18]. Some lesions may show blood flow, possibly due to tissue repair and vascular proliferation [19]. In this study, using BI‐RADS 4A or lower as benign, the ultrasound diagnostic accuracy was 58.2%, indicating that more than half of DM ultrasound findings lack malignant features such as solid echoes, structural distortion, significant mass effect, or rich blood flow. Unlike previous reports, this study suggests that DM ultrasound findings are nonmass lesions, and the analysis of internal echoes and structural characteristics is more important than traditional morphological analysis. The ultrasound findings were divided into four types; Type IV presented with slightly hyperechoic areas (2/67, or 3.0%). The Type IV slightly hyperechoic areas (2/67, 3.0%) being rare but previously reported in case studies [20]. One case was associated with thromboangiitis obliterans. Although the ultrasound diagnostic accuracy was slightly lower than mammography and MRI, the four characteristic types have significant value in suggesting DM.

Previous studies indicate that mammography findings are often nonspecific, showing focal asymmetric density without definite masses, calcifications, or structural distortion; nearly half (46.7%) of the lesions are negative [2, 21, 22]. Dense breasts may obscure DM, reducing detection sensitivity [23]. In this study, 18 lesions (31.0%) were negative on mammography, and 32 (55.2%) showed asymmetric density with no suspicious calcifications or structural distortion, consistent with previous studies. DM tends to be obvious on palpation but not on mammography or does not have typical signs of malignancy on mammography, and the diagnostic suggestiveness rate is slightly higher than that of ultrasound for DM in which lesions have been detected.

DM on MRI can present as nonmass lesions, isointense on T1WI, and isointense to slightly hypointense on T2WI, with nonspecific patchy or diffuse enhancement on dynamic contrast‐enhanced MRI. Typical findings include low early enhancement with gradual increase, showing a Type I TIC [24, 25]. DWI findings vary [25], with DM lesions composed of dense fibrous tissue and low cellularity generally showing no abnormally high signal and no decrease in ADC values. However, lesions with significant lymphocytic infiltration may show high signal and decreased ADC values. A few case reports suggest that DM on MRI may show malignant features, such as early significant enhancement, Type III TIC, and decreased ADC values, requiring differentiation from breast cancer. In this study, five lesions in five patients underwent MRI, showing benign features on hemodynamic parameters and DWI, with a diagnostic accuracy higher than ultrasound and mammography.

**Figure 4:**
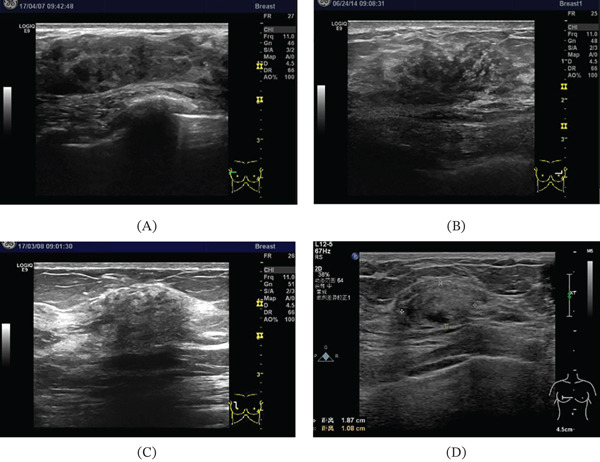
Typical illustrations of four ultrasound feature classifications in DM. (A) Type I, characterized by focal thickening and expansion of the gland along with mixed high and low internal echogenicity. (B) Type II, marked by focal or diffuse hypoechoic areas with indistinct margins. (C) Type III, distinguished by diffuse hypoechogenicity within the lesion, poorly visualized internal structures, and pronounced posterior acoustic shadowing. (D) Type IV, which presented with slightly hyperechoic areas.

In conclusion, if the multimodal imaging and clinical information of the lesion exhibit the following characteristics, it strongly suggests DM: ultrasound findings consistent with Types I, II, III, or IV (Figure 4), mammography showing negative or nonspecific focal density without high‐density masses, structural distortion, or suspicious calcifications, and MRI showing nonmass lesions with benign hemodynamic and DWI features, combined with clinical palpation and diabetes history. The above characteristics strongly suggest DM even without pathological examination.

Our finding further discusses and summarizes the multimodal imaging features highly suggestive of DM. Clinicians can analyze these features in combination with clinical information to potentially improve diagnostic accuracy, thereby minimizing the need for biopsies and reducing misdiagnosis. Our findings may help mitigate patients′ risks of infection, psychological, and financial burdens, while avoiding potential stimulation of lesions. Although invasive pathological examination remains the definitive diagnostic standard, our research holds promise for offering a clear line of reasoning and evidence to guide clinical diagnosis and treatment.

The limitation of this study is the small sample size, especially for MRI (only five cases). Future studies must expand the sample size for further analysis.

## Author Contributions

Yilin Xu and Yingxue Wang have contributed to the work equally and should be regarded as cofirst authors.

## Funding

This study was supported by the Tianjin Key Medical Discipline Construction Project (Grant No. TJYXZDXK‐3‐015C and TJYXZDXK‐3‐003A) and the National Natural Science Foundation of China (No. 82172025).

## Conflicts of Interest

The authors declare no conflicts of interest.

## Data Availability

The data that support the findings of this study are available on request from the corresponding author. The data are not publicly available due to privacy or ethical restrictions.
